# Postoperative endophthalmitis

**Published:** 2015

**Authors:** Nuwan Niyadurupola, Nick Astbury

**Affiliations:** Consultant Ophthalmic Surgeon: Norfolk and Norwich University Hospital, Norwich, UK. nuwan.niya@doctors.org; Senior Clinical Lecturer: International Centre for Eye Health, London School of Hygiene and Tropical Medicine, UK

**Figure:**
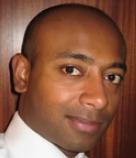
Nuwan Niyadurupola

**Figure:**
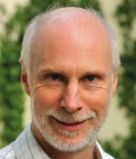
Nick Astbury

Endophthalmitis may have devastating consequences for a patient's vision and therefore should be treated as an emergency. The time from diagnosis to treatment is critical for favourable outcomes. In order to achieve a rapid response, it is important to have an accessible protocol and an endophthalmitis kit at hand for all eye surgeons who see postoperative patients. We have produced a simple protocol of recommended practice collated from a range of sources (see opposite page).

All intraocular procedures carry a risk of endophthalmitis, but – globally – they are most commonly reported following cataract surgery and intravitreal injections; this is due to the sheer numbers of both of these procedures carried out throughout the world. The prophylactic steps in the protocol have particular reference to cataract surgery, but similar practice should be adopted for any intraocular procedure. The clinical diagnosis and treatment is similar for all cases of endophthalmitis.

Careful preparation of the patient prior to performing an intraocular procedure is vitally important to reduce the risk of endophthalmitis.^1^ The patient should have 5% povidone iodine instilled into the conjunctival sac and the eye should be carefully draped to isolate the surgical field from the eyelids and the lashes.^1–5^ The eyelashes need **not** be cut as cutting the lashes does not reduce periocular bacterial flora and does not reduce the risk of endophthalmitis.^5^ The surgeon should wash his or her hands effectively and wear sterile gown and gloves.^1^ At the conclusion of cataract surgery, intracameral cefuroxime, if available, should be given to reduce the risk of endophthalmitis.^6^

The development of a red eye, pain and blurred vision in the days or weeks following an intraocular procedure should be considered as a case of endophthalmitis until proven otherwise. If intraocular inflammation is discovered, particularly if there is a hypopyon, treatment for endophthalmitis should be initiated without delay.

**NOTE: Do not try to treat with a course of corticosteroids first – this will delay treatment and may result in losing the eye.**

An endophthalmitis kit should be accessible in every practice where postoperative patients are seen and is invaluable to allow prompt diagnosis and treatment (see panel below). A vitreous biopsy/tap through the pars plana should be performed immediately for gram stain and culture. If the patient has perception of light only, a vitrectomy has been shown to be more beneficial than a vitreous tap.^7^ However, if a delay is likely before a vitrectomy can be performed, it is advisable to perform a vitreous tap and inject intravitreal antibiotics for more rapid treatment.

Intravitreal antibiotics (vancomycin and ceftazidime or amikacin and ceftazidime) should be given immediately, using separate syringes and needles for each drug (see panel for instructions to make up the required concentrations of each antibiotic). The use of intravitreal dexamethasone (a steroid) is controversial.

Consider adjunctive systemic therapy –with the same antibiotics as those used intravitreally – for 48 hours to maintain higher levels within the posterior segment of the eye. If systemic antibiotics are not available, topical antibiotics are better than nothing. Careful monitoring of the patient is important. The response to treatment and the results of gram stain and culture should determine whether further intravitreal antibiotic therapy is required.

**Contents of the Endophthalmitis Kit or Pack**

**Equipment for preparation of patient**

- Tetracaine (anaesthetic) drops
- Povidone iodine
- Drape
- Speculum
- Equipment for sub-Tenon's anaesthetic injection
  – 10 ml 2% lidocaine
  – 10 ml syringe
  – Sub-Tenon's cannula
  – Westcott scissors

**Equipment for vitreous biopsy/tap**

- 23 G or 25 G needle
- 5 ml syringe
- Calipers

**Equipment for preparation of antibiotic injections**

- 1 vial of 500 mg vancomycin or 1 vial of 500 mg (250 mg/ml) amikacin
- 1 vial of 500 mg ceftazidime
- 3 × 10 ml sodium chloride 0.9% injection (saline)
- 4 × 10 ml syringe
- 2 × 5 ml ^syringe
- 2 × 1 ml syringe
- 1 × sterile galley pot (for amikacin)
- 6 × 21 G needles for preparation of antibiotics
- 2 × 30 G needles for intravitreal injection

**Written instructions for preparation of antibiotic injections (to be prepared prior to vitreous tap and biopsy)**

- Vancomycin 1 mg/0.1 ml
  – Reconstitute 500 mg vial with 10 ml saline
  – Withdraw all 10 ml into 10 ml syringe
  – Inject 2 ml of this solution back into vial
  – Add 8 ml saline into vial to make up to 10 ml (10 mg/ml)
  – Use 1 ml syringe to draw 0.1 ml of this solution (1 mg/0.1 ml)
- Amikacin 400 μg/0.1 ml
  – Use 10 ml syringe to withdraw 1.6 ml of amikacin (250 mg/ml)
  – Make up to 10 ml in the syringe with saline
  – Discard 9 ml from syringe and make the remaining 1 ml up to 10 ml (in the syringe) with more saline
  – Transfer the solution into a sterile galley pot and use 1 ml syringe to draw 0.1 ml of this solution (400 μg/0.1 ml)
- Ceftazidime 2 mg/0.1 ml
  – Reconstitute 500 mg vial with 10 ml saline
  – Withdraw all 10 ml into 10 ml syringe
  – Inject 2 ml of this solution back into vial
  – Add 3 ml saline into vial to make up to 5 ml (20 mg/ml)
  – Use 1 ml syringe to draw 0.1 ml of this solution (2 mg/0.1 ml)
